# Intestinal Damages by F18^+^ *Escherichia coli* and Its Amelioration with an Antibacterial Bacitracin Fed to Nursery Pigs

**DOI:** 10.3390/antiox12051040

**Published:** 2023-05-03

**Authors:** Marcos Elias Duarte, Chad H. Stahl, Sung Woo Kim

**Affiliations:** 1 Department of Animal Science, North Carolina State University, Raleigh, NC 27695, USA; 2 Department of Animal and Avian Sciences, University of Maryland, College Park, MD 20742, USA

**Keywords:** F18^+^ *E. coli*, growth performance, intestinal health, oxidative damages, pigs

## Abstract

This study investigated intestinal oxidative damage caused by F18^+^ *Escherichia coli* and its amelioration with antibacterial bacitracin fed to nursery pigs. Thirty-six weaned pigs (6.31 ± 0.08 kg BW) were allotted in a randomized complete block design. Treatments were: NC, not challenged/not treated; PC, challenged (F18^+^ *E. coli* at 5.2 × 10^9^ CFU)/not treated; AGP challenged (F18^+^ *E. coli* at 5.2 × 10^9^ CFU)/treated with bacitracin (30 g/t). Overall, PC reduced (*p* < 0.05) average daily gain (ADG), gain to feed ratio (G:F), villus height, and villus height to crypt depth ratio (VH:CD), whereas AGP increased (*p* < 0.05) ADG, and G:F. PC increased (*p* < 0.05) fecal score, F18^+^ *E. coli* in feces, and protein carbonyl in jejunal mucosa. AGP reduced (*p* < 0.05) fecal score and F18^+^ *E. coli* in jejunal mucosa. PC reduced (*p* < 0.05) *Prevotella stercorea* populations in jejunal mucosa, whereas AGP increased (*p* < 0.05) *Phascolarctobacterium succinatutens* and reduced (*p* < 0.05) *Mitsuokella jalaludinii* populations in feces. Collectively, F18^+^ *E. coli* challenge increased fecal score and disrupted the microbiota composition, harming intestinal health by increasing oxidative stress, and damaging the intestinal epithelium, ultimately impairing growth performance. Dietary bacitracin reduced reduced F18^+^ *E. coli* populations and the oxidative damages they cause, thereby improving intestinal health and the growth performance of nursery pigs.

## 1. Introduction

In swine production, the post-weaning period is associated with immunological, physiological, psychological, and nutritional challenges that can impair the intestinal immune system and growth performance of pigs [1,2,3]. The impaired intestinal immune system increases pigs susceptibility to pathogen invasion [3,4]. Enterotoxigenic *Escherichia coli*, which causes post-weaning diarrhea (PWD), is a pathogen of concern for producers around the world. As a consequence of *E. coli* infection, changes in intestinal microbiota can led to increased inflammation and oxidative damage in the intestine, ultimately resulting in growth retardation [5,6,7]. According to Duarte and Kim [8], the changes in the intestinal microbiota in pigs challenged with F18^+^ *E. coli* are positively correlated with oxidative damages in the jejunal mucosa.

Different strategies have been utilized to reduce the susceptibility of pigs to potential pathogens [9,10]. Since the 1950s, antibiotics have been used in swine feed to promote growth by improving intestinal health [11,12]. Bacitracin is an antibiotic commonly used in animal feeds as a growth promoter and to treat and control infections [13]. In the US, bacitracin use as a growth promoter is not subjected to the veterinary feed directive rule and, therefore, does not require veterinary prescription [14]. Although the use of bacitracin has been primarily thought of as effective against Gram-positive pathogens, its use has also been reported to modulate the intestinal microbiota in nursery pigs [7], rabbits [15], and poultry [16,17]. This modulation of microbiota may explain the ability of bacitracin ability to prevent the deleterious effects of *E. coli* infection [7].

If the damage caused by F18^+^ *E. coli* infection is partially due to alterations in intestinal microbiota, which led to increased oxidative damage and increased intestinal inflammatory responses, understanding ways to mediate this is important for improving the efficiency of swine production. Bacitracin may be a useful tool to minimize the disruption of the intestinal microbiota due to F18^+^ *E. coli* infection, consequently promoting the growth of challenged pigs. To test this hypothesis, this study evaluated the intestinal oxidative damages caused by F18^+^ *E. coli* and its protection with the antibacterial bacitracin fed to nursery pigs.

## 2. Materials and Methods

The Institutional Animal Care and Use Committee at North Carolina State University approved the experimental protocol used in this study, as stated in the North Carolina State Animal Care and Use Procedures (REG 10.10.01).

### 2.1. Animals, Experimental Design, Diets, and Inoculation

An amount of 36 newly weaned pigs (18 barrows and 18 gilts) with 6.31 ± 0.08 kg body weight (BW) and 21 d of age were allotted to 3 treatments using a randomized complete block design (RCBD). Sex and initial BW were considered as blocks. The treatments were: NC, not challenged/not treated; PC, challenged (F18^+^ *E. coli* at 5.2 × 10^9^ CFU)/not treated; AGP, challenged (F18^+^ *E. coli* at 5.2 × 10^9^ CFU)/treated with bacitracin (30 g/t). Pigs were fed diets for 28 d divided into 2 phases (P1 for 14 d, and P2 for 14 d). Basal diets were formulated to meet the nutrient requirements suggested by NRC [18] (Table 1).

Bacitracin methylene disalicylate (BMD) was added to the diets as a source of bacitracin. After 7 d of feeding (pre-challenge period), all pigs on PC and AGP received an oral dose of F18^+^ *E. coli* (5.2 × 10^9^ CFU), and pigs on NC received an oral dose of sterile saline solution. The F18^+^ *E. coli* culture was prepared and inoculated to the challenged pigs, as previously reported by Duarte and Kim [8] and Xu et al. [7]. The inoculum was produced by utilizing the F18ac (O147) strain that generates heat-stable toxins A (STa) and B (STb). The strain stock was tested to confirm the expression of F18ac, STa, and STb.

### 2.2. Growth Performance and Fecal Score

Body weight and feed intake were measured weekly to calculate the average daily gain (ADG), average feed intake (ADFI), and the gain to feed ratio (G:F) in order to evaluate the growth performance of pigs. The fecal scores were recorded every other day using a scoring system where 1 = very hard and dry stool, 2 = firm stool; 3 = normal stool; 4 = loose stool; and 5 = watery stool, as previously reported by [19,20]

### 2.3. Sample Collection and Processing

Fecal and blood samples were collected from all pigs at d 14 and 28. Fecal samples were freshly collected to evaluate the microbiota composition in the post-challenge period. Blood (10 mL) was collected from the jugular vein into vacutainer tubes without anticoagulant to obtain serum to determine the concentration of tumor necrosis-alpha (TNF-α), as an indicator of inflammatory status [21] and protein carbonyl, as an indicator of oxidative stress status [22]. Sera were stored at −80 °C until analysis.

After 28 d feeding, all pigs were euthanized by penetrating captive bolt followed by exsanguination. Jejunal tissue and mucosa were collected 3 m distal to the pyloric-duodenal junction. Jejunal tissue (5 cm) was collected in 10% buffered formalin, and mucosa was obtained from the next 20 cm of jejunum and snap frozen in liquid nitrogen. The mucosa samples were used to evaluate the microbiota composition, the inflammatory and the oxidative stress status. Protein extracts from the mucosa were obtained by homogenization homogenizer (Tissuemiser; Fisher Scientific Inc., Waltham, MA, USA) in phosphate-buffered saline (PBS). The homogenate was then centrifuged at 10,000× *g* at 4 °C for 15 min, and the supernatant stored at −80 °C for further analysis.

### 2.4. Immune and Oxidative Stress Status

Protein concentration of samples were determined using the Protein Assay Kit (23225#, Thermo Fisher Scientific Inc., Wilmington, DE, USA). Prior to analysis, the samples were diluted in PBS at 1:80 and 1:40 for serum and mucosa samples, respectively. Concentrations of TNF-α in mucosa and protein carbonyl in mucosa and sera were normalized to total protein content, as previously reported by Cheng et al. [23]. The concentration of TNF-α was measured in serum and mucosa samples using the Porcine TNF-α Immunoassay Kit (#PTA00; R&D Systems, Minneapolis, MN, USA) as previously described by Holanda and Kim [24]. The concentration of protein carbonyl was measured using the OxiSelect Protein carbonyl ELISA Kit (Cell Biolabs, Inc., San Diego, CA, USA) as previously described by Jang et al. [25].

### 2.5. Intestinal Morphology and Crypt Cell Proliferation

Jejunal tissue samples were sent to the North Carolina State University Histology Laboratory (College of Veterinary Medicine, Raleigh, NC, USA) for Ki-67 staining [21]. Fifteen fields of view at 40× magnification of villi and their respective crypts per pig were used to measure villi height and width and crypt depth. The villi height to crypt depth ratio (VH:CD) was then calculated. Fifteen fields of view at 100× magnification were used to determine the proportion of Ki-67^+^ to total cells in the crypt as an estimator of cell proliferation rate in crypts, as previously described by Duarte and Kim [22].

### 2.6. Intestinal Microbiota

DNA was extracted (DNA Stool Mini Kit,#51604, Qiagen; Germantown, MD, USA) from fecal and mucosa samples for 16S rRNA analysis and for quantification of F18^+^ *E. coli* by qPCR. The DNA samples were sent to MAKO laboratories (Raleigh, NC, USA) for 16S rRNA and qPCR analysis according to their protocol, as reported by Duarte et al. [26]. The relative abundance of microbiota was calculated, and the OTU (operational taxonomic unit) with <0.5% relative abundance was combined and reported as “Others”.

The F18^+^ *E. coli* in the mucosa and fecal samples was quantified by qPCR following the protocol used by MAKO laboratories. Briefly, the plasmid containing the F18 fimbriae genes fedA (NCBI GeneBank, accession no. M61713) was constructed using the GeneArt (Thermo Fisher Scientific). The synthetic F18 gene was assembled from synthetic oligonucleotides. The fragment was inserted into the pMK-RQ-Bs vector GeneArt (Thermo Fisher Scientific). The concentration of plasmid DNA was measured by UV spectroscopy after the purification from the transformed bacteria. The similarity of sequence within the insertion sites was 100%. A TaqMan probe specific to the fedA gene was provided by Thermo Fisher. For quantification of F18^+^ plasmid in the samples, the assembled vector was used as standard.

The standard vector was linearized using the SmaI digestion (#FD0664, Thermo Fisher Scientific) prior to sequencing using qPCR. The count of the stock standard was calculated based on the vector size (914 bp). Then, the standard was diluted to 2.86 × 10^7^, 2.86 × 10^6^, 2.86 × 10^5^, 2.86 × 10^4^, and 2.86 × 10^3^. The Taqpath qPCR Master Mix CG (#A15297, Thermo Fisher Scientific) and the QuantStudio 12K Flex (Thermo Fisher Scientific) were used for the qPCR of samples and standards following the instructions of the manufacturer. Based on the count of the plasmid on the standard, linear regression was used to calculate the concentration of the F18^+^ plasmid in the samples. Before statistical analysis, the concentration of F18^+^ plasmid was Log transformed.

### 2.7. Statistical Analysis

The Mixed procedure of SAS 9.4 Software (Cary, NC, USA) was used to analyze all data based on a randomized complete block design. The main effect was the treatments, and the random effects were sex and initial BW. Pre-planned contrasts were used to test the effect of the F18^+^ *E. coli* challenge (NC vs. PC) and the effect of AGP on challenged pigs (PC vs. AGP). Statistical differences were considered significant with *p* < 0.05, and the tendency was considered when 0.05 ≤ *p* < 0.10.

## 3. Results

### 3.1. Growth Performance and Fecal Score

Prior to challenge (d 0 to 7), the treatments did not affect BW, ADG, ADFI, or G:F (Table 2). After the *E. coli* challenge, the PC had lower (*p* < 0.05) BW at d 14, 21, and 28 when compared with the NC. The AGP-treated pigs had higher (*p* < 0.05) BW at d 14 and tended to have higher BW (*p* = 0.066) at d 28 when compared with PC. The PC reduced (*p* < 0.05) the ADG of pigs post-challenge (d7 to 14, d 14 to 21, and d 7 to 28) and over the entire experiment (d 0 to 28) when compared to the NC. The AGP increased (*p* < 0.05) the ADG of pigs post-challenge (d 14 to 21, and d 7 to 28) and over the entire experiment (d 0 to 28) when compared with PC. During the last week of the experiment, d 21 to 28, the treatments did not affect the ADG, ADFI, nor the G:F. The PC did not affect the ADFI during the entire experiment, whereas AGP tended to increase ADFI (*p* = 0.073) from d 7 to 14. The PC reduced (*p* < 0.05) the G:F of pigs during the post-challenge (d7 to 14, and d 7 to 28) and the overall experiment (d 0 to 28) when compared with NC. The AGP increased (*p* < 0.05) the G:F of pigs, compared to the PC, from d 14 to 21.

**Figure 1:**
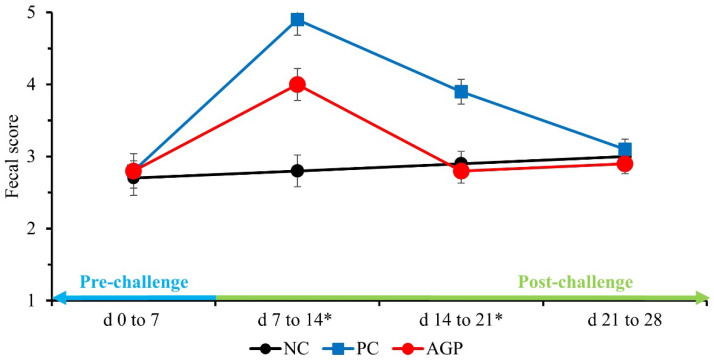
The fecal score of pigs challenged with F18^+^ *Escherichia coli* and fed diets with bacitracin as a growth promoter. NC, not challenged/not treated; PC, challenged (*E. coli* F18^+^ at 5.2 × 10^9^ CFU)/not treated; AGP, challenged (*E. coli* F18^+^ at 5.2 × 10^9^ CFU)/treated with bacitracin (30 g/t). * d 7 to 14: NC vs. PC: (*p* = 0.001), PC vs. AGP: (*p* = 0.004); d 14 to 21: NC vs. PC: (*p* = 0.001), PC vs. AGP: (*p* = 0.001).

Before the *E. coli* challenge (d 0 to 7), the treatments did not affect the fecal score of pigs (Figure 1). After the challenge, the PC pigs had higher (*p* < 0.05) fecal scores during the first- and second-week post-challenge when compared with NC. The AGP pigs had fecal scores that were intermediate to the PC and the NC during the first week post-challenge (*p* < 0.05), and they were not significantly different than those of the NC during the second week post-challenge. There were no significant differences in fecal score among the treatments during the final week of the experiment.

### 3.2. F18^+^ E. coli Counting

**Figure 2:**
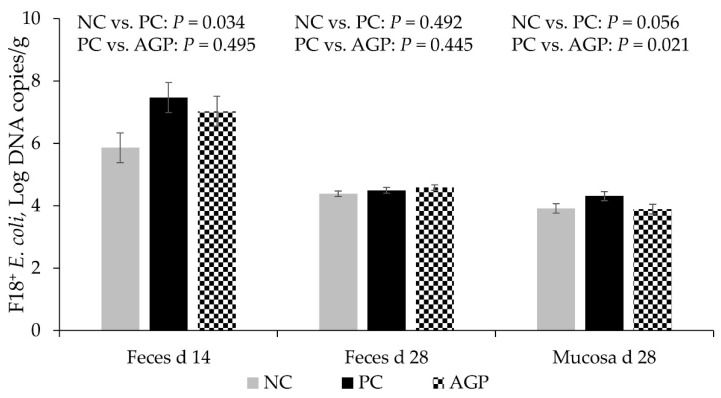
F18^+^ *E. coli* counting in feces and jejunal mucosa of pigs challenged with F18^+^ *Escherichia coli* and fed diets with bacitracin as a growth promoter. NC, not challenged/not treated; PC, challenged (*E. coli* F18^+^ at 5.2 × 10^9^ CFU)/not treated; AGP, challenged (*E. coli* F18^+^ at 5.2 × 10^9^ CFU)/treated with bacitracin (30 g/t).

The PC had increased (*p* < 0.05) copies of fedA, indicating higher populations of F18^+^ *E. coli* in the feces of pigs at d 14 when compared with the NC. There are no significant differences in feces at d 28 (Figure 2). The AGP did not significantly impact the copies of fedA in the feces on d 14. The PC tended to have greater (*p* = 0.056) concentrations of fedA in samples from jejunal mucosa, compared to the NC at d 28, and AGP appeared to have concentrations that were significantly lower than those of the PC group.

### 3.3. Immune and Oxidative Stress Status

The concentration of TNF-α in jejunal mucosa was not affected by the treatments (Table 3). The PC tended to increase the concentration of TNF-α in sera at d 14 when compared with NC. The PC increased (*p* < 0.05) the concentration of protein carbonyl in serum and jejunal mucosa at d 28 when compared with NC. The AGP tended to reduce (*p* < 0.05) the concentration of protein carbonyl in the jejunal mucosa of pigs at d 28 when compared with PC.

### 3.4. Intestinal Morphology and Cell Proliferation in Crypt

The PC reduced (*p* < 0.05) the villus height and the VH:CD in the jejunum of pigs when compared with NC (Table 4). The villus width, crypt depth, and cell proliferation in jejunal crypts were not affected by the treatments.

### 3.5. Relative Abundance and Diversity of the Fecal and Mucosa-Associated Microbiota

The PC reduced (*p* < 0.05) the relative abundance of Tenericutes and tended to reduce (*p* = 0.095) the relative abundance of Deferribacteres in the feces of pigs at d 28 when compared with NC (Table 5). The PC tended to increase (*p* = 0.072) the relative abundance of Firmicutes in the feces of pigs at d 28 when compared with NC. The AGP tended to increase (*p* = 0.088) the relative abundance of Actinobacteria in the feces of pigs at d 28 when compared with PC. The PC tended to reduce (*p* = 0.055) the relative abundance of Bacteroidetes in the jejunal mucosa of pigs when compared with NC.

The PC increased (*p* < 0.05) the relative abundance of *Lachnospiraceae* and tended to increase (*p* = 0.072) the relative abundance of *Campylobacteraceae* in the feces of pigs at d 14 when compared with NC (Table 6). The AGP tended to reduce (*p* = 0.094) the relative abundance of Others in the feces of pigs at d 14 when compared with PC. The PC tended to increase (*p* = 0.099) the relative abundance of *Acidaminococcaceae* in the jejunal mucosa of pigs when compared with NC (Table 7). The AGP did not affect the relative abundance of mucosa-associated microbiota at the family level in the jejunum of pigs.

The PC tended to reduce (*p* = 0.079) the relative abundance of *Succinivibrio dextrinosolvens* in the feces of pigs at d 14 when compared with NC (Table 8). The PC tended to reduce (*p* = 0.065) the relative abundance of *Prevotella stercorea* and increased the relative abundance of *Mitsuokella jalaludinii* in the feces of pigs at d 28 when compared with NC. The AGP increased (*p* < 0.05) the relative abundance of *Phascolarctobacterium succinatutens* whereas reduced (*p* < 0.05) the relative abundance of *Mitsuokella jalaludinii* in feces of pigs at d 28 when compared with PC.

The PC tended to reduce the relative abundance of *Prevotella copri* (*p* = 0.090), *Phascolarctobacterium succinatutens* (*p* = 0.053), and *Lactobacillus delbrueckii* (*p* = 0.050) in jejunal mucosa of pigs when compared with NC (Table 9). The AGP did not affect the relative abundance of mucosa-associated microbiota in pigs challenged with F18^+^ *E. coli*.

**Figure 3:**
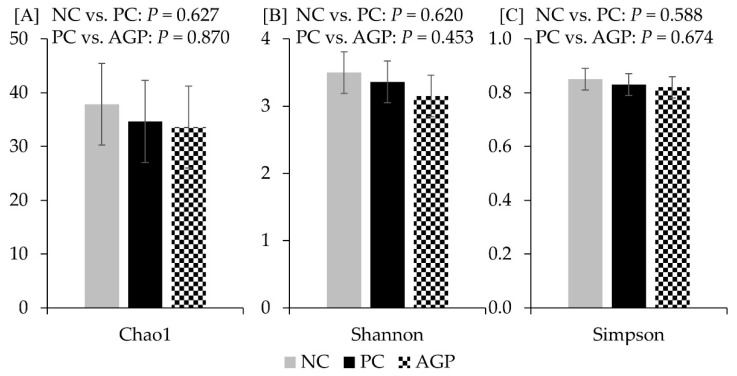
Alpha diversity of fecal microbiota at d 14 estimated with Chao1 richness (**A**), Shannon diversity (**B**), and Simpson diversity (**C**) in pigs challenged with F18^+^ *Escherichia coli* and fed diets with bacitracin as a growth promoter. NC, not challenged/not treated; PC, challenged (*E. coli* F18^+^ at 5.2 × 10^9^ CFU)/not treated; AGP, challenged (*E. coli* F18^+^ at 5.2 × 10^9^ CFU)/treated with bacitracin (30 g/t).

**Figure 4:**
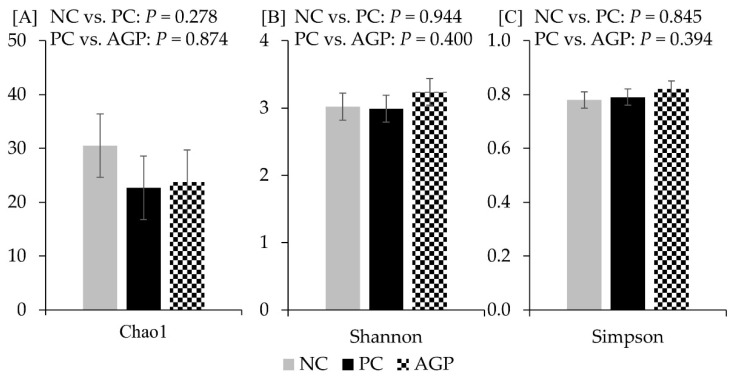
Alpha diversity of fecal microbiota at d 28 estimated with Chao1 richness (**A**), Shannon diversity (**B**), and Simpson diversity (**C**) in pigs challenged with F18^+^ *Escherichia coli* and fed diets with bacitracin as a growth promoter. NC, not challenged/not treated; PC, challenged (*E. coli* F18^+^ at 5.2 × 10^9^ CFU)/not treated; AGP, challenged (*E. coli* F18^+^ at 5.2 × 10^9^ CFU)/treated with bacitracin (30 g/t).

**Figure 5:**
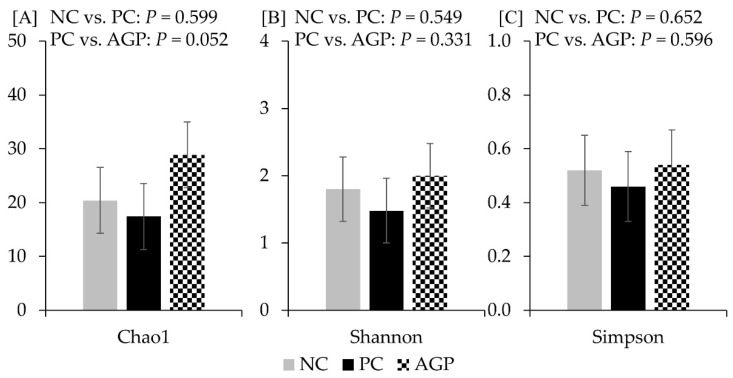
Alpha diversity of mucosa-associated microbiota at d 28 estimated with Chao1 richness (**A**), Shannon diversity (**B**), and Simpson diversity (**C**) in pigs challenged with F18^+^ *Escherichia coli* and fed diets with bacitracin as a growth promoter. NC, not challenged/not treated; PC, challenged (*E. coli* F18^+^ at 5.2 × 10^9^ CFU)/not treated; AGP, challenged (*E. coli* F18^+^ at 5.2 × 10^9^ CFU)/treated with bacitracin (30 g/t).

The alpha diversity of fecal microbiota was not affected by the treatments at d 14 and d 28 (Figure 3 and Figure 4). However, AGP tended to increase (*p* = 0.052) the alpha diversity of mucosa-associated microbiota estimated with Chao1 (Figure 5).

## 4. Discussion

In this study, direct oral challenge with F18^+^ *E. coli* caused PWD, increased oxidative damage, and reduced the growth performance of weaned pigs, which is in agreement with previous reports [7,27]. The reduced feed efficiency seen among the *E. coli* challenged pigs can be attributed to the impaired intestinal health of challenged pigs as observed by increased fecal score, increased inflammation and oxidative stress, and the damaged villi and disrupted microbial community. The health challenged pigs may have had reduced nutrient absorption and/or altered partitioning of nutrients for immune response and growth, resulting in reduced feed efficiency [5,28]. However, bacitracin ameliorated many of the effects of the *E. coli* challenge, as evidenced by improved fecal scores, reduced oxidative damage and improved the feed efficiency. These benefits were seen without a significant reduction in fecal shedding of *E. coli* post-challenge.

Changes in diet, environment, social interaction, and the removal of the passive immunity from sow’s milk during a period where the immune system is not fully mature increase the vulnerability of newly weaned pigs to opportunistic pathogens [1,10,29]. The F18^+^ *E. coli* attaches to glycoproteins on the brush border in the intestine mediating resistance to flushing and promoting colonization [30,31,32]. In the current study, the increased F18^+^ *E. coli* counting in feces at d 14 matches with the increased fecal score in the period of 7 to 14 d of the experiment and may be an indicator of proliferation on the intestinal epithelium. At d 28, 21 d after challenge, the F18^+^ *E. coli* counting in feces did not differ among treatments, and fecal scores returned to normal, indicating pigs had controlled the *E. coli* infection to a less harmful level. The trend toward increased F18^+^ *E. coli* in the jejunal mucosa of challenged pigs indicates that F18^+^ *E. coli* can persist in the gastrointestinal tract for up to 21 d post-challenge. Duarte and Kim [8] reported that F18^+^ *E. coli* has a long-lasting effect in jejunal mucosa when compared with feces.

Interestingly, bacitracin reduced the F18^+^ *E. coli* population in jejunal mucosa. Antibiotics have been used to overcome or mitigate the challenges associated with health and nutrition, mainly by impairing the growth of pathogens [11,12]. Bacitracin, produced by *Bacillus licheniformis*, is an antibiotic with a narrow spectrum against primarily Gram-positive bacteria [33]. Bacitracin inhibits the synthesis of peptidoglycan and teichoic acids in the cell wall of bacteria inhibiting their proliferation [34,35]. Gram-positive bacteria are the main target for bacitracin due to the thicker peptidoglycan layer [35,36]. However, Gram-negative bacteria also contain peptidoglycan on the cell wall [37]. Xu et al. [7] demonstrated that bacitracin can mitigate the effects of PWD caused by F18^+^ *E. coli* in nursery pigs.

During proliferation, *E. coli* can produce enterotoxins, including STa and STb, that induce the secretion of fluid in the lumen of the small intestine, causing diarrhea [7,30]. Pigs challenged with F18^+^ *E. coli* in this study had increased fecal scores until d 21 of the experiment. Challenged pigs that received bacitracin showed improved fecal scores at d 14, although they remained higher than those of the unchallenged pigs. By d 21, the fecal scores of the F18^+^ *E. coli* challenged pigs treated with bacitracin were similar to those of the unchallenged controls. These results demonstrate the efficacy of bacitracin in mitigating PWD in pigs, as previously reported by Xu et al. [7]. Duarte and Kim [8] reported that, although the diarrhea symptoms ceased 14 d after an F18^+^ *E. coli*-challenge, the effects of F18^+^ *E. coli* on intestinal health lasted for at least 21 d. In addition to diarrhea, F18^+^ *E. coli* infections can also result in an inflammatory response [7,8]. A systemic inflammatory response was seen 14 d after challenge in this study, with a trend toward increased concentration of TNF-α in the serum of challenged pigs. However, there were no differences at d 28 and in TNF-α concentrations in jejunal mucosa. Other studies have reported increased expression of IL-6 and IL-8 in the jejunal mucosa of F18^+^ *E. coli*-challenged pigs without a significant change in TNF-α expression [7,38]. Due to the complex timing of cytokine cascades during an immune response, it is not necessarily surprising that TNF-α concentrations in the intestinal mucosa were not elevated at the end of the study. At the completion of the study, sera and mucosal concentrations of protein carbonyl were increased in the challenged pigs. These findings are in agreement with previous works that have reported that a F18^+^ *E. coli* challenge increases oxidative stress in nursery pigs [5,7,27]. During infection, ROS, including nitrite, are produced by immune cells to fight the infection [39,40,41]. The antioxidant enzymes scavenge the ROS maintaining homeostasis [40]. When the production of ROS exceeds the antioxidant capacity, products from oxidative stress, including protein carbonyls, are generated [42]. Protein carbonyl has been reported as an important biomarker of oxidative stress because it can be produced by all ROS, and it has higher stability compared with other products of oxidative damage [43]. Protein carbonyls lead to the dysfunction of cellular proteins, which can induce apoptosis [41,44]. In this study, bacitracin treatment tended to reduce protein carbonyl concentrations in challenged pigs, possibly by altering gut microbiota and reducing the intestinal mucosa’s immunoreaction in response to the F18^+^ *E. coli* or by altering the production of toxins and other antigens by the *E. coli* [45].

The altered fluid secretion induced by enterotoxins from *E. coli* can reduce water absorption and increase the flux of water from the enterocyte into the lumen of the intestine, causing dehydration and cell apoptosis [46,47,48]. Previous studies have shown that the apoptosis induced by cell dehydration and oxidative damage in challenged pigs is associated with the reduction in villus length [46,49]. In this study, pigs challenged with F18^+^ *E. coli* had the lower villus height in jejunum, confirming the deleterious effects of the *E. coli* on the epithelium. Enterocyte damage in the villi can induce cell proliferation in crypts to provide new enterocytes [49]. Increased cell proliferation can increase crypt depth, therefore reducing the villus height to crypt depth ratio [50,51,52], which was seen with *E. coli* challenge in this study. According to Pluske et al. [50], the atrophy of villi and the hyperplasia of crypts can reduce the digestion and absorption of nutrients, thereby reducing the feed efficiency of pigs. Additionally, undigested nutrients can further contribute to PWD due to the increased amount of substrate available for microbial fermentation [6,53].

Increased fluid secretion, products from an immune response, and undigested nutrients can all modulate the microbiome toward a more inflammatory microbiota, such as increasing the abundance of Proteobacteria [5,6,7,8]. This change in the microbiota composition is associated with the increased production of ROS, including nitrite, released during the immune response. The nitrite is transformed into nitrate in the lumen favoring the growth of bacteria expressing nitrate reductase, such as Proteobacteria [5,54]. However, 7 d after the challenge, there was a trend of increasing Firmicutes on the feces of pigs mainly by increasing *Lachnospiraceae* while reducing Tenericutes and Deferribacteries. The environmental changes near the mucosa may have exerted pressure on the microbiota, moving *Lachnospiraceae* toward the luminal content, consequently modulating the luminal and the mucosa-associated microbiota [6,8]. Additionally, it was observed a trend towards increasing *Campylobacteraceae* in the feces of challenged pigs. Interestingly, at 21 d after the challenge, the abundance of was increased in the feces of challenged pigs. According to Duarte and Kim [17], *Mitsuokella* spp. and *Campylobacter* spp. are highly correlated to inflammatory and oxidative stress in pigs challenged with F18^+^ *E. coli*.

According to Belkaid et al. [55], the immune system plays a pivotal role in modulating the mucosa-associated microbiota, which in turn modulate the luminal microbiota. The relative abundance of *Prevotella* spp. and *Phascolarctobacterium succinatutens* in jejunal mucosa-associated microbiota was reduced in challenged pigs, possibly due to the oxidative environment promoted by the immune response against *E. coli*. *Prevotella* is a Bacteroidetes that is associated with health conditions, and its relative abundance increases in pigs after weaning due to the fiber content in the diet [6,56]. The unbalance in the microbiota composition by reducing the abundance of fiber-degrading bacteria, in turn, can increase the immune response in the intestine [5,6]. Interestingly, bacitracin tended to increase the alpha diversity of mucosa-associated microbiota in the jejunum. Previous studies have demonstrated that bacitracin can increase microbial diversity [7,17,57], although, in general, antibiotics are associated with reduced diversity [58]. According to Proctor and Phillips [17], the bacitracin may have inhibited the proliferation of certain bacteria allowing the growth of others. These effects were observed in fecal samples at d 28, where the abundance of *P. succinatutens* was increased, and *M. jalaludinii* populations were reduced. *M. jalaludinii* are Gram-negative bacteria confirming that bacitracin can also affect bacteria other than Gram-positive. *Phascolarctobacterium succinatutens*, a strict anaerobic bacteria belonging to Firmicutes, are associated with propionate production through the succinate scavenge [59,60]. Succinate is normally produced by different bacteria within the intestine, especially from carbohydrate fermentation [60]. It has been reported that succinate exerts inflammatory [61] and oxidative [62] roles. Therefore, these findings suggest that the reduction in protein carbonyl reported in the current study can also be associated with the increased abundance of bacteria associated with fiber utilization, including *P. succinatutens*.

## 5. Conclusions

The F18^+^ *E. coli* challenge resulted in increased fecal scores, altered intestinal histology, increased oxidative damage, all demonstrating reduced intestinal health. This resulted in impaired growth performance of pigs challenged with F18^+^ *E. coli*. Dietary supplementation with bacitracin ameliorates many of the intestinal health challenges caused by F18^+^ *E. coli,* resulting in improved growth performance. Whereas further studies are needed to elucidate the protective mechanisms of bacitracin on a F18^+^ *E. coli* infection, alterations in the microbiota towards a less harmful milieu may underlay this effect and ultimately provide greater insight into the role of microbiota on improving growth performance.

## Figures and Tables

**Table 1:** Composition of basal diets (Exp. 1; as-fed basis).

Item	Phase 1	Phase 2
Ingredient, %		
Corn, yellow	40.45	54.47
Soybean meal, 48% CP	22.00	23.50
Whey permeate	20.00	10.00
Blood plasma	6.00	3.00
Poultry meal	5.00	4.00
Poultry fat	3.50	1.80
_L_-Lys HCl	0.48	0.47
DL-Met	0.22	0.18
_L_-Thr	0.15	0.13
_L_-Trp	0.00	0.00
Dicalcium phosphate	0.60	0.85
Limestone	0.95	0.95
Vitamin premix ^1^	0.03	0.03
Mineral premix ^2^	0.15	0.15
Salt	0.22	0.22
Zinc oxide	0.25	0.25
Calculated composition:		
Dry matter, %	90.8	90.1
ME, kcal/kg	3481	3388
CP, %	23.00	21.60
SID ^3^ Lys, %	1.50	1.35
SID Met + Cys, %	0.82	0.74
SID Trp, %	0.25	0.22
SID Thr, %	0.88	0.79
Ca, %	0.86	0.81
STTD ^4^ *p*, %	0.45	0.40
Total *p*, %	0.67	0.64

^1^ The vitamin premix provided the following per kilogram of complete diet: 6613.8 IU of vitamin A as vitamin A acetate, 992.0 IU of vitamin D3, 19.8 IU of vitamin E, 2.64 mg of vitamin K as menadione sodium bisulfate, 0.03 mg of vitamin B12, 4.63 mg of riboflavin, 18.52 mg of D-pantothenic acid as calcium pantothenate, 24.96 mg of niacin, and 0.07 mg of biotin. ^2^ The trace mineral premix provided the following per kilogram of complete diet: 4.0 mg of Mn as manganous oxide, 165 mg of Fe as ferrous sulfate, 165 mg of Zn as zinc sulfate, 16.5 mg of Cu as copper sulfate, 0.30 mg of I as ethylenediamine di-hydroiodide, and 0.30 mg of Se as sodium selenite. ^3^ SID, standardized ileal digestible. ^4^ STTD, standardized total tract digestible.

**Table 2:** Growth performance of pigs challenged with F18^+^ *Escherichia coli* and fed diets with bacitracin as a growth promoter.

	Treatment ^1^				*p* Value	
Item	NC	PC	AGP	SEM	NC vs. PC	PC vs. AGP
BW, kg						
Initial	6.31	6.31	6.30	0.08	0.985	0.912
d 7	6.91	6.90	6.93	0.15	0.958	0.852
d 14	8.64	7.72	8.20	0.28	0.036	0.231
d 21	11.93	10.21	11.88	0.46	0.019	0.018
d 28	16.25	14.19	15.94	0.64	0.040	0.066
ADG, kg						
Pre-challenge (d 0 to 7)	0.080	0.084	0.091	0.021	0.980	0.804
Post-challenge (d 7 to 28)	0.445	0.348	0.429	0.026	0.020	0.039
d 7 to 14	0.247	0.118	0.181	0.028	0.005	0.119
d 14 to 21	0.470	0.356	0.526	0.035	0.038	0.003
d 21 to 28	0.617	0.569	0.580	0.042	0.430	0.857
Overall	0.353	0.282	0.344	0.022	0.022	0.046
ADFI, kg						
Pre-challenge (d 0 to 7)	0.120	0.149	0.142	0.024	0.415	0.844
Post-challenge (d 7 to 28)	0.643	0.582	0.663	0.048	0.403	0.254
d 7 to 14	0.340	0.303	0.386	0.034	0.436	0.073
d 14 to 21	0.627	0.572	0.681	0.064	0.467	0.149
d 21 to 28	0.972	0.870	0.921	0.071	0.300	0.589
Overall	0.512	0.474	0.532	0.038	0.497	0.285
G:F						
Pre-challenge (d 0 to 7)	0.61	0.55	0.57	0.10	0.679	0.845
Post-challenge (d 7 to 28)	0.72	0.56	0.62	0.03	0.001	0.121
d 7 to 14	0.76	0.36	0.46	0.07	0.001	0.276
d 14 to 21	0.77	0.65	0.78	0.04	0.060	0.036
d 21 to 28	0.64	0.67	0.63	0.03	0.634	0.378
Overall	0.71	0.55	0.61	0.03	0.009	0.236

^1^ NC, not challenged/not treated; PC, challenged (*E. coli* F18^+^ at 5.2 × 10^9^ CFU)/not treated; AGP, challenged (*E. coli* F18^+^ at 5.2 × 10^9^ CFU)/treated with bacitracin (30 g/t).

**Table 3:** Immune and oxidative stress status of pigs challenged with F18^+^ *Escherichia coli* and fed diets with bacitracin as a growth promoter.

	Treatment ^1^				*p* Value	
Item	NC	PC	AGP	SEM	NC vs. PC	PC vs. AGP
Tumor necrosis factor-alpha						
d 14 serum, pg/mL	110.4	128.7	119.4	6.3	0.054	0.365
d 28 serum, pg/mL	114.7	107.9	104.8	8.6	0.586	0.796
Jejunal mucosa, pg/mg protein	1.31	1.66	1.76	0.26	0.361	0.773
Protein carbonyl, nmol/mg protein						
d 14 serum	2.20	2.08	2.16	0.11	0.419	0.605
d 28 serum	1.63	2.37	2.32	0.19	0.010	0.851
Jejunal mucosa	2.15	3.61	2.56	0.35	0.012	0.059

^1^ NC, not challenged/not treated; PC, challenged (*E. coli* F18^+^ at 5.2 × 10^9^ CFU)/not treated; AGP, challenged (*E. coli* F18^+^ at 5.2 × 10^9^ CFU)/treated with bacitracin (30 g/t).

**Table 4:** Intestinal morphology and cell proliferation in crypts of pigs challenged with F18^+^ *Escherichia coli* and fed diets with bacitracin as a growth promoter.

	Treatment ^1^				*p* Value	
Item	NC	PC	AGP	SEM	NC vs. PC	PC vs. AGP
Villus height, µm	527	394	436	32	0.003	0.296
Villus width, µm	85	91	85	7	0.378	0.419
Crypt depth, µm	245	253	253	12	0.591	0.975
VH:CD ^2^	2.22	1.58	1.73	0.14	0.002	0.398
Ki-67^+, 2^ (%)	52.0	47.3	51.6	5.3	0.473	0.511

^1^ NC, not challenged/not treated; PC, challenged (*E. coli* F18^+^ at 5.2 × 10^9^ CFU)/not treated; AGP, challenged (*E. coli* F18^+^ at 5.2 × 10^9^ CFU)/treated with bacitracin (30 g/t). ^2^ Cell proliferation rate.

**Table 5:** Relative abundance of fecal and mucosa-associated microbiota at the phylum level in pigs challenged with F18^+^ *Escherichia coli* and fed diets with bacitracin as a growth promoter.

	Treatment ^1^				*p* Value	
Item	NC	PC	AGP	SEM	NC vs. PC	PC vs. AGP
d 14 (Feces)						
Bacteroidetes	41.3	37.2	39.3	3.3	0.380	0.651
Firmicutes	36.2	42.9	45.6	5.1	0.375	0.705
Proteobacteria	19.6	15.4	11.6	5.9	0.629	0.653
Spirochaetes	2.1	3.2	2.3	1.3	0.571	0.619
Tenericutes	0.4	1.0	0.4	0.3	0.170	0.146
Actinobacteria	0.1	0.1	0.6	0.2	0.878	0.121
Deferribacteres	0.1	0.2	0.1	0.1	0.628	0.337
d 28 (Feces)						
Firmicutes	46.1	53.9	55.7	2.8	0.072	0.649
Bacteroidetes	41.2	38.8	37.1	2.2	0.458	0.595
Proteobacteria	10.1	5.8	5.2	2.5	0.234	0.855
Tenericutes	1.4	0.1	0.2	0.4	0.043	0.906
Deferribacteres	0.5	0.0	0.0	0.2	0.095	0.912
Spirochaetes	0.4	0.7	0.6	0.4	0.653	0.865
Actinobacteria	0.1	0.5	1.2	0.3	0.438	0.088
d 28 (Mucosa)						
Firmicutes	50.5	46.2	51.0	14.6	0.839	0.821
Proteobacteria	25.9	43.1	39.3	15.6	0.452	0.867
Bacteroidetes	22.0	9.6	8.9	4.3	0.055	0.904
Actinobacteria	0.8	0.8	0.4	0.5	0.946	0.571
Spirochaetes	0.4	0.2	0.3	0.2	0.367	0.463

^1^ NC, not challenged/not treated; PC, challenged (*E. coli* F18^+^ at 5.2 × 10^9^ CFU)/not treated; AGP, challenged (*E. coli* F18^+^ at 5.2 × 10^9^ CFU)/treated with bacitracin (30 g/t).

**Table 6:** Relative abundance of fecal microbiota at the family level in pigs challenged with F18^+^ *Escherichia coli* and fed diets with bacitracin as a growth promoter.

	Treatment ^1^				*p* Value	
Item	NC	PC	AGP	SEM	NC vs. PC	PC vs. AGP
d 14						
*Prevotellaceae*	29.6	29.0	26.6	3.4	0.874	0.571
*Veillonellaceae*	10.8	10.9	14.2	4.3	0.989	0.599
*Enterobacteriaceae*	8.9	6.5	5.8	5.5	0.764	0.926
*Ruminococcaceae*	7.0	6.1	6.1	2.2	0.767	0.977
*Acidaminococcaceae*	2.5	4.5	6.2	1.6	0.402	0.480
*Porphyromonadaceae*	4.4	4.4	7.8	2.1	0.978	0.266
*Lactobacillaceae*	1.7	4.2	7.3	2.2	0.449	0.339
*Lachnospiraceae*	3.0	5.1	4.4	0.7	0.049	0.469
*Succinivibrionaceae*	6.6	3.6	2.9	2.0	0.309	0.825
*Eubacteriaceae*	2.0	2.8	3.0	0.5	0.301	0.813
*Clostridiaceae*	2.3	3.3	2.2	1.2	0.576	0.544
*Cytophagaceae*	3.3	1.3	0.9	1.3	0.302	0.813
*Campylobacteraceae*	0.6	3.7	1.9	1.2	0.072	0.284
*Erysipelotrichaceae*	1.2	1.3	1.3	0.6	0.919	0.984
*Spirochaetaceae*	1.4	1.3	1.4	1.1	0.983	0.967
*Rikenellaceae*	1.9	0.3	1.5	1.1	0.310	0.453
*Peptococcaceae*	2.6	0.9	0.1	1.4	0.414	0.709
*Bacteroidaceae*	0.6	1.4	1.8	0.6	0.368	0.627
Others	9.6	9.6	5.0	1.8	0.995	0.094
d 28						
*Prevotellaceae*	36.6	33.5	30.8	2.5	0.407	0.456
*Veillonellaceae*	21.0	27.1	21.1	2.8	0.144	0.150
*Lactobacillaceae*	7.7	4.6	3.6	2.7	0.438	0.793
*Ruminococcaceae*	4.5	5.0	7.2	0.9	0.737	0.121
*Lachnospiraceae*	3.5	4.9	6.4	1.4	0.404	0.544
*Acidaminococcaceae*	3.4	5.1	6.3	1.4	0.390	0.533
*Porphyromonadaceae*	2.5	2.6	4.5	0.9	0.922	0.189
*Succinivibrionaceae*	5.2	2.7	2.2	2.3	0.450	0.882
*Eubacteriaceae*	1.8	2.3	3.3	0.4	0.423	0.132
*Streptococcaceae*	0.9	1.3	4.2	1.6	0.864	0.226
*Clostridiaceae*	1.4	1.5	1.5	0.4	0.772	0.949
*Campylobacteraceae*	2.3	1.1	0.4	0.8	0.312	0.567
Others	9.2	8.2	8.5	1.9	0.703	0.904

^1^ NC, not challenged/not treated; PC, challenged (*E. coli* F18^+^ at 5.2 × 10^9^ CFU)/not treated; AGP, challenged (*E. coli* F18^+^ at 5.2 × 10^9^ CFU)/treated with bacitracin (30 g/t).

**Table 7:** Relative abundance of mucosa-associated microbiota at the family level in pigs challenged with F18^+^ *Escherichia coli* and fed diets with bacitracin as a growth promoter.

	Treatment ^1^				*p* Value	
Item	NC	PC	AGP	SEM	NC vs. PC	PC vs. AGP
*Lactobacillaceae*	25.6	23.8	21.4	12.7	0.922	0.893
*Helicobacteraceae*	14.6	35.6	34.7	15.7	0.365	0.968
*Prevotellaceae*	19.6	8.9	8.4	4.5	0.117	0.938
*Veillonellaceae*	7.6	5.38	19.2	5.7	0.763	0.115
*Streptococcaceae*	9.4	12.1	6.3	6.7	0.778	0.551
*Campylobacteraceae*	6.5	6.0	2.6	4.7	0.948	0.615
*Acidaminococcaceae*	2.6	1.0	0.8	0.7	0.099	0.862
*Enterobacteriaceae*	4.2	1.7	2.2	1.1	0.128	0.766
*Lachnospiraceae*	2.1	0.8	1.2	0.6	0.151	0.675
*Ruminococcaceae*	1.2	0.6	0.8	0.3	0.233	0.691
*Erysipelotrichaceae*	0.4	1.3	0.2	0.6	0.379	0.271
*Clostridiaceae*	0.4	0.6	0.2	0.2	0.556	0.268
*Bifidobacteriaceae*	0.6	0.7	0.3	0.4	0.897	0.539
*Porphyromonadaceae*	1.3	0.4	0.4	0.4	0.129	0.929
*Succinivibrionaceae*	0.7	0.5	1.0	0.5	0.749	0.479
Others	4.2	1.7	2.2	1.1	0.128	0.766

^1^ NC, not challenged/not treated; PC, challenged (*E. coli* F18^+^ at 5.2 × 10^9^ CFU)/not treated; AGP, challenged (*E. coli* F18^+^ at 5.2 × 10^9^ CFU)/treated with bacitracin (30 g/t).

**Table 8:** Relative abundance of fecal microbiota at the specie level in pigs challenged with F18^+^ *Escherichia coli* and fed diets with bacitracin as a growth promoter.

	Treatment ^1^				*p* Value	
Item	NC	PC	AGP	SEM	NC vs. PC	PC vs. AGP
d 14						
*Prevotella copri*	36.8	29.7	20.1	5.6	0.376	0.238
*Phascolarctobacterium succinatutens*	4.8	8.8	13.2	3.8	0.468	0.427
*Prevotella stercorea*	10.2	6.8	9.4	3.4	0.482	0.582
*Faecalibacterium prausnitzii*	10.1	8.0	9.1	3.2	0.648	0.811
*Succinivibrio dextrinosolvens*	6.8	3.0	0.7	1.5	0.079	0.288
*Dialister succinatiphilus*	2.5	1.6	6.3	3.0	0.845	0.290
*Roseburia faecis*	3.7	3.1	2.1	1.3	0.723	0.598
*Campylobacter coli*	1.1	4.8	6.0	2.4	0.275	0.720
*Prevotella* sp.	3.0	1.4	2.7	1.3	0.388	0.495
*Mitsuokella jalaludinii*	2.8	2.3	3.9	1.2	0.783	0.364
*Brachyspira hampsonii*	1.8	3.0	2.6	1.5	0.605	0.877
*Treponema porcinum*	0.9	1.8	3.7	1.2	0.594	0.262
*Campylobacter lanienae*	0.6	3.0	3.2	1.3	0.214	0.924
*Dorea longicatena*	0.4	0.9	1.2	0.4	0.410	0.641
*Lactobacillus mucosae*	0.5	0.4	0.4	1.2	0.939	0.985
*Mitsuokella multacida*	0.3	1.5	1.8	0.6	0.159	0.675
*Lactobacillus kitasatonis*	0.3	1.1	0.6	0.5	0.290	0.542
*Gemmiger formicilis*	1.7	1.2	0.6	0.6	0.585	0.472
Others	12.0	17.9	12.5	3.6	0.261	0.308
d 28						
*Prevotella copri*	39.0	35.2	30.3	6.0	0.656	0.568
*Prevotella stercorea*	9.3	5.0	8.6	1.6	0.065	0.119
*Dialister succinatiphilus*	4.2	8.0	3.2	3.2	0.409	0.294
*Phascolarctobacterium succinatutens*	4.3	3.0	10.0	2.0	0.658	0.024
*Faecalibacterium prausnitzii*	5.2	4.0	7.3	1.5	0.551	0.121
*Mitsuokella jalaludinii*	3.3	8.7	2.0	1.6	0.025	0.007
*Roseburia faecis*	3.1	3.6	3.6	1.1	0.754	0.992
*Prevotella* sp.	2.2	4.5	2.2	1.4	0.262	0.259
*Streptococcus alactolyticus*	1.0	1.6	4.5	2.6	0.867	0.435
*Succinivibrio dextrinosolvens*	4.4	2.3	1.8	2.2	0.502	0.876
*Lactobacillus delbrueckii*	4.4	1.1	0.5	2.6	0.373	0.873
*Lactobacillus kitasatonis*	1.7	0.7	0.8	1.5	0.654	0.958
*Acidaminococcus fermentans*	1.4	3.9	1.4	1.1	0.110	0.108
*Gemmiger formicilis*	1.1	1.2	1.6	0.8	0.901	0.699
*Mitsuokella multacida*	0.7	2.3	1.5	0.8	0.194	0.523
*Campylobacter coli*	2.7	0.8	0.5	0.8	0.127	0.837
*Selenomonas bovis*	1.6	1.3	2.2	0.9	0.797	0.489
*Lactobacillus mucosae*	1.4	1.3	0.6	0.7	0.911	0.496
*Dorea longicatena*	0.2	1.3	4.4	1.5	0.595	0.168
*Megasphaera hominis*	1.5	0.9	1.3	0.4	0.367	0.568
*Selenomonas lipolytica*	0.9	1.4	2.2	0.8	0.637	0.476
Others	6.4	7.9	9.5	1.9	0.578	0.574

^1^ NC, not challenged/not treated; PC, challenged (*E. coli* F18^+^ at 5.2 × 10^9^ CFU)/not treated; AGP, challenged (*E. coli* F18^+^ at 5.2 × 10^9^ CFU)/treated with bacitracin (30 g/t).

**Table 9:** Relative abundance of mucosa-associated microbiota at the specie level in pigs challenged with F18^+^ *Escherichia coli* and fed diets with bacitacin as growth promoter.

	Treatment ^1^				*p* Value	
Item	NC	PC	AGP	SEM	NC vs. PC	PC vs. AGP
*Helicobacter rappini*	7.3	26.3	14.2	11.1	0.240	0.449
*Helicobacter mastomyrinus*	7.5	11.2	22.4	7.6	0.732	0.311
*Lactobacillus kitasatonis*	9.9	10.4	5.4	7.2	0.959	0.631
*Lactobacillus mucosae*	9.7	4.5	11.3	7.4	0.627	0.526
*Prevotella copri*	17.8	9.3	12.1	3.4	0.090	0.560
*Streptococcus alactolyticus*	10.4	10.1	4.7	4.5	0.967	0.408
*Campylobacter upsaliensis*	2.8	3.8	2.6	5.7	0.905	0.879
*Streptococcus infantarius*	3.2	5.4	3.9	2.6	0.549	0.679
*Dialister succinatiphilus*	2.9	1.9	2.9	1.2	0.555	0.568
*Prevotella stercorea*	5.1	1.5	1.2	1.1	0.036	0.853
*Phascolarctobacterium succinatutens*	4.8	1.0	0.8	1.3	0.053	0.937
*Lactobacillus salivarius*	0.7	0.9	1.2	1.7	0.945	0.906
*Faecalibacterium prausnitzii*	1.7	0.8	1.4	0.7	0.346	0.524
*Lactobacillus delbrueckii*	2.2	0.6	0.9	0.6	0.050	0.751
*Helicobacter* sp.	1.3	0.0	0.0	1.2	0.473	0.992
*Mitsuokella jalaludinii*	0.7	1.4	1.8	0.9	0.600	0.788
Others	12.0	11.0	13.5	4.6	0.875	0.705

^1^ NC, not challenged/not treated; PC, challenged (*E. coli* F18^+^ at 5.2 × 10^9^ CFU)/not treated; AGP, challenged (*E. coli* F18^+^ at 5.2 × 10^9^ CFU)/treated with bacitracin (30 g/t).

## Data Availability

The data presented in this study are available in the article.

## References

[1] McLamb B.L., Gibson A.J., Overman E.L., Stahl C., Moeser A.J. (2013). Early weaning stress in pigs impairs innate mucosal immune responses to enterotoxigenic *E. coli* challenge and exacerbates intestinal injury and clinical disease. PLoS ONE.

[2] Campbell J.M., Crenshaw J.D., Polo J. (2013). The biological stress of early weaned piglets. J. Anim. Sci. Biotechnol..

[3] Moeser A.J., Pohl C.S., Rajput M. (2017). Weaning stress and gastrointestinal barrier development: Implications for lifelong gut health in pigs. Anim. Nutr..

[4] Sun Y., Kim S.W. (2017). Intestinal challenge with enterotoxigenic *Escherichia coli* in pigs, and nutritional intervention to prevent postweaning diarrhea. Anim. Nutr..

[5] Duarte M.E., Tyus J., Kim S.W. (2020). Synbiotic effects of enzyme and probiotics on intestinal health and growth of newly weaned pigs challenged with enterotoxigenic F18^+^
*Escherichia coli*. Front. Vet. Sci..

[6] Duarte M.E., Kim S.W. (2022). Intestinal microbiota and its interaction to intestinal health in nursery pigs. Anim. Nutr..

[7] Xu X., Duarte M.E., Kim S.W. (2022). Postbiotic effects of Lactobacillus fermentate on intestinal health, mucosa-associated microbiota, and growth efficiency of nursery pigs challenged with F18^+^
*Escherichia coli*. J. Anim. Sci..

[8] Duarte M.E., Kim S.W. (2022). Significance of mucosa-associated microbiota and its impacts on intestinal health of pigs challenged with F18^+^
*E. coli*. Pathogens.

[9] Jang K.B., Purvis J.M., Kim S.W. (2021). Dose–response and functional role of whey permeate as a source of lactose and milk oligosaccharides on intestinal health and growth of nursery pigs. J. Anim. Sci..

[10] Zheng L., Duarte M.E., Sevarolli Loftus A., Kim S.W. (2021). Intestinal health of pigs upon weaning: Challenges and nutritional intervention. Front. Vet. Sci..

[11] Carpenter L.E. (1951). The effect of antibiotics and vitamin B12 on the growth of swine. Arch. Biochem. Biophys..

[12] Luecke R.W., Thorp F., Newland H.W., Mcmillen W.N. (1951). The growth promoting effects of various antibiotics on pigs. J. Anim. Sci..

[13] Bridges J.H., Hale F., Kunkel H.O., Lyman C.M. (1954). The effects of bacitracin, penicillin and arsanilic acid on growth rate and feed efficiency in swine. J. Anim. Sci..

[14] FDA Food and Drug Administration (2013). Guidance for Industry #213: New Animal Drugs and New Animal Drug Combination Products Administered in or on Medicated Feed or Drinking Water of Food- Producing Animals: Recommendations for Drug Sponsors for Voluntarily Aligning Product Use Conditions with.

[15] Chen Y., Hu S., Li J., Zhao B., Yang N., Zhou T., Liang S., Bai S., Wu X. (2021). Bacitracin methylene disalicylate improves intestinal health by modulating its development and microbiota in weaned rabbits. Front. Microbiol..

[16] Johnson T.A., Sylte M.J., Looft T. (2019). In-feed bacitracin methylene disalicylate modulates the turkey microbiota and metabolome in a dose-dependent manner. Sci. Rep..

[17] Proctor A., Phillips G.J. (2019). Differential effects of bacitracin methylene disalicylate (BMD) on the distal colon and cecal microbiota of young broiler chickens. Front. Vet. Sci..

[18] NRC (2012). Nutrient Requirements of Swine.

[19] Deng Z., Duarte M.E., Jang K.B., Kim S.W. (2022). Soy protein concentrate replacing animal protein supplements and its impacts on intestinal immune status, intestinal oxidative stress status, nutrient digestibility, mucosa-associated microbiota, and growth performance of nursery pigs. J. Anim. Sci..

[20] Jang K.B., Duarte M.E., Purvis J.M., Kim S.W. (2021). Impacts of weaning age on dietary needs of whey permeate for pigs at 7 to 11 kg body weight. J. Anim. Sci. Biotechnol..

[21] Moita V.H.C., Duarte M.E., Kim S.W. (2022). Functional roles of xylanase enhancing intestinal health and growth performance of nursery pigs by reducing the digesta viscosity and modulating the mucosa-associated microbiota in the jejunum. J. Anim. Sci..

[22] Duarte M.E., Kim S.W. (2022). Phytobiotics from oregano extracts enhance the intestinal health and growth performance of pigs. Antioxidants.

[23] Cheng Y.-C., Duarte M.E., Kim S.W. (2022). Effects of Yarrowia lipolytica supplementation on growth performance, intestinal health and apparent ileal digestibility of diets fed to nursery pigs. Anim. Biosci..

[24] Holanda D.M., Kim S.W. (2021). Investigation of the efficacy of mycotoxin-detoxifying additive on health and growth of newly-weaned pigs under deoxynivalenol challenges. Anim. Biosci..

[25] Jang K.B., Kim J.H., Purvis J.M., Chen J., Ren P., Vazquez-Anon M., Kim S.W. (2020). Effects of mineral methionine hydroxy analog chelate in sow diets on epigenetic modification and growth of progeny. J. Anim. Sci..

[26] Duarte M.E., Sparks C., Kim S.W. (2021). Modulation of jejunal mucosa-associated microbiota in relation to intestinal health and nutrient digestibility in pigs by supplementation of β-glucanase to corn–soybean meal-based diets with xylanase. J. Anim. Sci..

[27] Jang K.B., Moita V.H.C., Martinez N., Sokale A., Kim S.W. (2023). Efficacy of zinc glycinate reducing zinc oxide on intestinal health and growth of nursery pigs challenged with F18^+^
*Escherichia coli*. J. Anim. Sci..

[28] Huntley N.F., Nyachoti C.M., Patience J.F. (2018). Lipopolysaccharide immune stimulation but not β-mannanase supplementation affects maintenance energy requirements in young weaned pigs. J. Anim. Sci. Biotechnol..

[29] Kim S.W., Duarte M.E. (2021). Understanding intestinal health in nursery pigs and the relevant nutritional strategies. Anim. Biosci..

[30] Dubreuil J.D., Isaacson R.E., Schifferli D.M. (2016). Animal enterotoxigenic *Escherichia coli*. EcoSal Plus.

[31] Peterson J.W., Whipp S.C. (1995). Comparison of the mechanisms of action of cholera toxin and the heat-stable enterotoxins of *Escherichia coli*. Infect. Immun..

[32] Nagy B., Whipp S.C., Imberechts H., Bertschinger H.U., Dean-Nystrom E.A., Casey T.A., Salajka E. (1997). Biological relationship between F18ab and F18ac fimbriae of enterotoxigenic and verotoxigenic *Escherichia coli* from weaned pigs with oedema disease or diarrhoea. Microb. Pathog..

[33] Huyghebaert G., De Groote G. (1997). The bioefficacy of zinc bacitracin in practical diets for broilers and laying hens. Poult. Sci..

[34] Tay W.M., Epperson J.D., da Silva G.F.Z., Ming L.-J. (2010). H NMR, Mechanism, and Mononuclear oxidative activity of the antibiotic metallopeptide bacitracin: The Role of d -Glu-4, Interaction with Pyrophosphate Moiety, DNA Binding and Cleavage, and Bioactivity. J. Am. Chem. Soc..

[35] Mascher T., Margulis N.G., Wang T., Ye R.W., Helmann J.D. (2003). Cell wall stress responses in Bacillus subtilis: The regulatory network of the bacitracin stimulon. Mol. Microbiol..

[36] Rajagopal M., Walker S. (2017). Envelope structures of Gram-positive bacteria. Current Topics in Microbiology and Immunology.

[37] Cheng Y.-C., Kim S.W. (2022). Use of microorganisms as nutritional and functional feedstuffs for nursery pigs and broilers. Animals.

[38] Wong B.T., Park S., Kovanda L., He Y., Kim K., Xu S., Lingga C., Hejna M., Wall E., Sripathy R. (2022). Dietary supplementation of botanical blends enhanced performance and disease resistance of weaned pigs experimentally infected with enterotoxigenic *Escherichia coli* F18. J. Anim. Sci..

[39] Yang Y., Bazhin A.V., Werner J., Karakhanova S. (2013). Reactive oxygen species in the immune system. Int. Rev. Immunol..

[40] Morris G., Gevezova M., Sarafian V., Maes M. (2022). Redox regulation of the immune response. Cell. Mol. Immunol..

[41] Celi P., Gabai G. (2015). Oxidant/antioxidant balance in animal nutrition and health: The role of protein oxidation. Front. Vet. Sci..

[42] Schieber M., Chandel N.S. (2014). ROS function in redox signaling and oxidative stress. Curr. Biol..

[43] Dalle-Donne I., Rossi R., Giustarini D., Milzani A., Colombo R. (2003). Protein carbonyl groups as biomarkers of oxidative stress. Clin. Chim. Acta.

[44] DalleDonne I., Milzani A., Colombo R. (1999). The tert -butyl hydroperoxide-induced oxidation of actin cys-374 is coupled with structural changes in distant regions of the protein. Biochemistry.

[45] Settle T., Leonard S.S., Falkenstei E., Fix N., Van Dyke K., Klandorf H. (2014). Effects of a phytogenic feed additive versus an antibiotic feed additive on oxidative stress in broiler chicks and a possible mechanism determined by electron spin resonance. Int. J. Poult. Sci..

[46] Johnson A.M., Kaushik R.S., Rotella N.J., Hardwidge P.R. (2009). Enterotoxigenic *Escherichia coli* modulates host intestinal cell membrane asymmetry and metabolic activity. Infect. Immun..

[47] Dubreuil J.D. (2017). Enterotoxigenic *Escherichia coli* and probiotics in swine: What the bleep do we know?. Biosci. Microbiota Food Health.

[48] Kennedy D.J., Greenberg R.N., Dunn J.A., Abernathy R., Ryerse J.S., Guerrant R.L. (1984). Effects of *Escherichia coli* heat-stable enterotoxin STb on intestines of mice, rats, rabbits, and piglets. Infect. Immun..

[49] Assimakopoulos S.F., Tsamandas A.C., Louvros E., Vagianos C.E., Nikolopoulou V.N., Thomopoulos K.C., Charonis A., Scopa C.D. (2011). Intestinal epithelial cell proliferation, apoptosis and expression of tight junction proteins in patients with obstructive jaundice. Eur. J. Clin. Investig..

[50] Pluske J.R., Hampson D.J., Williams I.H. (1997). Factors influencing the structure and function of the small intestine in the weaned pig: A review. Livest. Prod. Sci..

[51] Shaw D. (2012). Intestinal mucosal atrophy and adaptation. World J. Gastroenterol..

[52] Pluske J.R., Williams I.H., Aherne F.X. (1996). Maintenance of villous height and crypt depth in piglets by providing continuous nutrition after weaning. Anim. Sci..

[53] Kim K., He Y., Xiong X., Ehrlich A., Li X., Raybould H., Atwill E.R., Maga E.A., Jørgensen J., Liu Y. (2019). Dietary supplementation of Bacillus subtilis influenced intestinal health of weaned pigs experimentally infected with a pathogenic *E. coli*. J. Anim. Sci. Biotechnol..

[54] Bäumler A.J., Sperandio V. (2016). Interactions between the microbiota and pathogenic bacteria in the gut. Nature.

[55] Belkaid Y., Hand T.W. (2014). Role of the microbiota in immunity and inflammation. Cell.

[56] Kovatcheva-Datchary P., Nilsson A., Akrami R., Lee Y.S., De Vadder F., Arora T., Hallen A., Martens E., Björck I., Bäckhed F. (2015). Dietary Fiber-induced improvement in glucose metabolism is associated with increased abundance of Prevotella. Cell Metab..

[57] Díaz Carrasco J.M., Redondo E.A., Pin Viso N.D., Redondo L.M., Farber M.D., Fernández Miyakawa M.E. (2018). Tannins and bacitracin differentially modulate gut microbiota of broiler chickens. Biomed Res. Int..

[58] Grazul H., Kanda L.L., Gondek D. (2016). Impact of probiotic supplements on microbiome diversity following antibiotic treatment of mice. Gut Microbes.

[59] Ikeyama N., Murakami T., Toyoda A., Mori H., Iino T., Ohkuma M., Sakamoto M. (2020). Microbial interaction between the succinate-utilizing bacterium Phascolarctobacterium faecium and the gut commensal Bacteroides thetaiotaomicron. Microbiologyopen.

[60] Fernández-Veledo S., Vendrell J. (2019). Gut microbiota-derived succinate: Friend or foe in human metabolic diseases?. Rev. Endocr. Metab. Disord..

[61] Tannahill G.M., Curtis A.M., Adamik J., Palsson-McDermott E.M., McGettrick A.F., Goel G., Frezza C., Bernard N.J., Kelly B., Foley N.H. (2013). Succinate is an inflammatory signal that induces IL-1β through HIF-1α. Nature.

[62] Chouchani E.T., Pell V.R., Gaude E., Aksentijević D., Sundier S.Y., Robb E.L., Logan A., Nadtochiy S.M., Ord E.N.J., Smith A.C. (2014). Ischaemic accumulation of succinate controls reperfusion injury through mitochondrial ROS. Nature.

